# The Investigation of Latanoprost's Effects on the Intraocular Pressure of Healthy Male guinea Pigs Under Light and Dark Regimes

**DOI:** 10.1002/vms3.70560

**Published:** 2025-08-22

**Authors:** Arghavan Armin, Farnoosh Arfaee

**Affiliations:** ^1^ Department of Clinical Sciences Faculty of Veterinary Medicine, The Science and Research Branch, Islamic Azad University Tehran Iran

**Keywords:** glaucoma | guinea pig | intraocular pressure | latanoprost | light | ocular hypertension

## Abstract

**Purpose:**

The current study aimed to investigate the effect of latanoprost on intraocular pressure (IOP) fluctuations of guinea pigs (*Cavia porcellus*) under different light and darkness regimes.

**Methods:**

16 healthy adult American pigmented male guinea pigs (*C. porcellus*) were used for this study. A single drop of 0.005% latanoprost ophthalmic solution (Lataprost® Sina Daru, Tehran, Iran) was administered in the right eye of guinea pigs at 5:30, whereas the left eye received a placebo. The guinea pigs were restrained gently by the experimenter while avoiding extra pressure on the neck and eyelids. Then, the animals were randomly divided into two groups (Groups A and B). Although Group A (*n* = 8) was exposed to 12 + 0.5 h of light, Group B (*n* = 8) was kept in a dark room throughout the whole experiment. IOP measurements were performed using rebound tonometry (TonoVet®, iCare; Helsinki, Finland) at 6:00, 7:00, 8:00 and 9:00, followed by measurements at 12:00, 15:00 and 18:00. Statistical analyses were conducted using repeated measures analysis of variance (ANOVA), but due to the small sample size and non‐normal data distribution, non‐parametric methods were employed, including the Wilcoxon Signed‐Rank Test for pre‐ and post‐treatment comparisons and the Mann‐Whitney *U* Test for between‐group differences (*p*‐values <0.05 were considered significant).

**Results:**

The IOP reduction in the treated eyes was greater in the guinea pigs in the dark after latanoprost instillation at 7:00 and 8:00 (−2.75 ± 1.49 and −1.37 ± 0.92 mmHg, respectively, *p*‐value <0.05) compared to those in the light (−0.62 ± 2.13 and −0.37 ± 1.92 mmHg, *p*‐value >0.05). Significant IOP reduction was also observed in the untreated eyes due to the systemic absorption of latanoprost.

**Conclusion:**

The reduction in IOP caused by 0.005% latanoprost was more pronounced in guinea pigs kept in darkness compared to the animal exposed to light, suggesting that the diurnal and nocturnal patterns of IOP fluctuations are clinically significant in ocular hypertension therapy and, thus, should be considered when applying ophthalmic hypotensive agents. The study of IOP in guinea pigs provides valuable insights into circadian variations in IOP, which could help optimize treatment protocols by tailoring latanoprost administration to align with peak efficacy periods, potentially improving glaucoma management and therapeutic outcomes.

## Graphical abstract

Latanoprost (0.005%) lowers intraocular pressure (IOP) more effectively in guinea pigs kept in darkness than in light. IOP reduction was significant at 7:00 and 8:00, with systemic absorption affecting untreated eyes. These results suggest that aligning treatment with circadian IOP variations may improve glaucoma management.

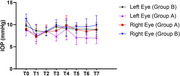

## 1 Introduction

Glaucoma refers to a group of ocular neuropathies associated with the loss of retinal ganglion cells and the degeneration of the optic nerve. This condition is often manifested by ocular hypotension due to impaired aqueous humour drainage and can lead to irreversible vision loss if left untreated (Komáromy et al. 2019; Weinreb et al. 2014). Currently, the application of intraocular pressure (IOP)‐reducing medications is the only effective solution for glaucoma management and blindness prevention (Ruangvaravate et al. 2020). The interspecies differences in the ocular anatomy and associated structures among different mammalian species account for the variations in the efficacy of the IOP‐lowering agents (Ofri 2002).

Although the guinea pig has a collagenous lamina cribrosa that is oriented radially, the protein concentration of its lamina cribrosa is comparable to that of humans and non‐human primates, making it a viable model for human ocular diseases such as myopia and glaucoma (Ostrin and Wildsoet 2016). Latanoprost, a prostaglandin analogue, has previously been shown to be effective in IOP reduction in guinea pigs by increasing the uveoscleral outflow of aqueous humour (Di et al. 2017).

Although exposure to light among several other environmental factors has been shown in previous literature to influence the 24 h pattern of IOP fluctuations and the regulation of circadian rhythm in mammalian species (Buijs and Kalsbeek 2001; Cahill and Menaker 1989; R. Y. Moore and Silver 1998), IOP regulation can be maintained in the absence of photic inputs, suggesting the involvement of an endogenous component (Bertolucci et al. 2009).

Given that glaucoma patients may experience a greater range of circadian IOP variation compared to the general population (Drance 1963), the establishment of circadian IOP patterns serves as an advantage in the diagnosis of early glaucoma.

This study aims to evaluate the effect of 0.005% latanoprost ophthalmic solution on IOP in guinea pigs, with a specific focus on how its efficacy varies under different lighting conditions (light and darkness). By assessing circadian IOP fluctuations and the pharmacological response to latanoprost in a controlled light‐dark cycle, this research seeks to determine whether guinea pigs exhibit a differential IOP‐lowering response based on environmental lighting. Given the structural and physiological similarities of the guinea pig eye to the human ocular system, particularly the presence of a collagenous lamina cribrosa, this study may provide valuable insights into the application of prostaglandin analogues in glaucoma management.

## 2 Materials and Methods

All guidelines regarding the work with laboratory animals have been observed during the experiment and animal handling. The ethics committee of the Science and Research Branch of the Islamic Azad University (IR.IAU.SRB.REC.1399.078) and the ARVO Statement for the Use of Animals in Ophthalmic and Vision Research approved the current study. A total of 16 healthy adult American pigmented male guinea pigs (*Cavia porcellus*) with an average weight of 550 g were obtained from the Laboratory Breeding Center of Pasteur Medical Research Institute of Iran. The animals were 12–18 months old at the time of the experiment and were further examined for possible ocular abnormalities by a veterinarian. A complete ophthalmologic examination was carried out on each animal, which consisted of fluorescein staining (Fluorescein Glostrips® Nomax Inc, St. Louis, USA), Schirmer tear test (Schirmer‐Tranetest Vet; Eickemeyer, Tuttlingen, Germany), slit‐lamp biomicroscopy (Kowa SL‐15; Kowa, Tokyo, Japan), direct (WA11710 Ophthalmoscope; Welch Allyn Inc, New York, USA) and indirect ophthalmoscopy (Binocular Indirect Ophthalmoscope; Welch Allyn Inc, New York, USA). Rebound tonometry (TonoVet®, iCare; Helsinki, Finland) was instrumented to ensure normal IOP. No ocular abnormalities were observed in any of the guinea pigs before the experiment.

Two weeks prior to the experiment, the animals were housed in individual boxes (0.5 m × 0.5 m) with white pine shaving bedding to acclimatize to the new light/darkness conditions (12 h of light followed by 12 h of darkness) (Devlin and Kay 2001; Reppert and Weaver 2002) and to recover from shipping‐related anxiety.

To ensure consistent experimental conditions when measuring IOP in guinea pigs, environmental factors such as noise levels, temperature stability and light uniformity were carefully controlled. The experiments were conducted in a quiet laboratory setting without background noise to minimize stress‐induced variations in IOP. The laboratory temperature was maintained within a controlled range (22–24°C), and fluctuations in temperature were minimized by avoiding direct exposure to air vents or drafts.

The light condition was provided by white 40‐W and yellow 9‐W LED lamps in the study room (3 m × 4 m) with no windows. The light source was carefully regulated to prevent fluctuations that could influence pupil size and IOP readings. Light exposure was standardized across all measurements to avoid glare or uneven lighting that could affect the accuracy of the readings.

Water was ad liberum at all times before and during the experiment, and there were no water or food restrictions. Due to the effect of metabolic signals feedback on the modulation of circadian rhythms (Green et al. 2008), a similar diet was provided for all animals, consisting of fresh fruits and vegetables along with guinea pig commercial pallets. Because of the influence of anaesthetic drugs on blood pressure and heart rate and its impact on IOP (Blumberg et al. 2007), the experiment was conducted without anaesthesia.

To collect IOP values, the animals were placed on a clean examination table to provide comfort and stability. The same restraining method was used for all animals, as different physical restraint techniques can significantly influence IOP readings, potentially affecting the accuracy and comparability of results across studies (Okur et al. 2023).

One hand gently supported the guinea pig's head, whereas the other held the tonometer, ensuring proper alignment with the cornea with no extra pressure applied on the neck and eyes to minimize changes in the respiratory and cardiovascular systems that would lead to false IOP elevation (Teng et al. 2003). The guinea pig's natural posture was maintained, and its body remained relaxed to prevent stress‐induced variability. To minimize stress, animals were acclimated to handling before the procedure, and measurements were taken in a quiet, dimly lit environment.

The guinea pigs were randomly divided into two groups to ensure unbiased group allocation and minimize selection bias. For this purpose, a web‐based randomization tool (Random.org) was used to build a randomized list, and each animal was given a unique number between 1 and 16. The first eight numbers in the randomized list were assigned to Group A, and the remaining eight numbers were assigned to Group B, each containing eight animals.

The animals in Group A were exposed to light from 5:30 to 18:00, whereas those in Group B were separated and transferred to another room with the same environmental conditions. The baseline right and left eye IOP values were measured at 5:30 in both groups under normal light conditions using the TonoVet (TonoVet®, iCare; Helsinki, Finland) ‘P’ mode for small rodents according to the device manual. A single drop of 0.005% preservative containing latanoprost ophthalmic solution (Lataprost®; Sina Daru, Tehran, Iran) was applied to the right eye of all animals at 5:30, whereas sterile artificial teardrop (Tearlose®; Sina Daru, Tehran, Iran) was instilled to the left eye as placebo.

After the drop administration at 5:30, the animals in Group B were kept in darkness for the whole duration of the experiment until 18:00. The IOP measurements during the dark phase were performed in the same room using dim red light applied from the room corner to minimize error (Aihara et al. 2003; Liu et al. 1999). The next IOP measurements of the right and left eyes were performed after 30 min at 6:00 and after 1 h at 7:00, 8:00 and 9:00, followed by measurements with 3 h intervals at 12:00, 15:00 and 18:00 in both groups. To eliminate individual bias, all data collection was done by a single experimenter.

## 3 Statistical Analysis

Repeated measure analysis of variance (ANOVA) test using SPSS statistical software (IBM, version 22) was used for all statistical analyses in this study. The arithmetic mean values (X) and standard deviation (SD) of the IOP were calculated and reported for each eye separately and at each measurement point after the administration of 0.005% latanoprost ophthalmic solution. Kolmogorov–Smirnov test was carried out to test the normal distribution of data.

Non‐parametric methods were employed in this study due to the small sample size (*n* = 8 per group) and the non‐normal distribution of IOP measurements, as confirmed by the Shapiro–Wilk test. For the right eye, the data was not normally distributed at T2, T5, T6 and T7 measurement points, whereas the same was true for the left eye at T2 and T5. Given that parametric tests require normally distributed data and may be unreliable with small sample sizes, non‐parametric alternatives were chosen to ensure robust and valid statistical inferences.

To assess differences in IOP changes between the light‐exposed (Group A) and darkness (Group B) conditions, the Mann–Whitney *U* test was applied, as it does not assume normality and is appropriate for independent groups with small sample sizes. Additionally, to analyse IOP variations across multiple time points within each group, Wilcoxon pairwise comparisons with Bonferroni correction were performed when significant differences were detected. By using these non‐parametric methods, we ensured that the analysis remained statistically rigorous despite the limitations posed by the data distribution and sample size, allowing for a reliable evaluation of the effect of latanoprost on IOP under different light conditions. *p*‐values less than 0.05 were considered to be statistically significant.

## 4 Results

The baseline (at 5:30) mean IOP ± SD values were lower in the right and left eyes in Group A (9.00 ± 1.41 and 8.75 ± 1.28 mmHg, respectively) compared to Group B (11.25 ± 1.16 and 9.75 ± 1.03 mmHg, respectively). The mean ± SD IOP values, as well as the median and range values associated with the right and left eyes in Groups A and B are reported in Table 1.

**TABLE 1:** The effect of 0.005% latanoprost on intraocular pressure (IOP) (mmHg) (mean values ± standard deviation [SD]) on the guinea pigs’ right and left eyes in Groups A and B during the 12 + 0.5 h of the experiment.

Group B	Left eye	Range	8.00–11.00	6.00–10.00	7.00–9.00	8.00–11.00	6.00–12.00	7.00–10.00	7.00–10.00	6.00–11.00
		Median	10.00	9.00	9.00	9.50	9.50	9.00	9.00	9.00
		Mean IOP ± SD (mmHg)	9.75 ± 1.03	8.62 ± 1.30	8.37 ± 0.92	9.50 ± 0.93	9.12 ± 1.81	9.00 ± 0.93	9.00 ± 1.07	8.87 ± 1.55
	Right eye	Range	10.00–13.00	6.00–9.00	7.00–10.00	8.00–12.00	7.00–12.00	8.00–11.00	7.00–13.00	7.00–14.00
		Median	11.00	7.00	8.50	10.00	9.00	9.50	9.00	9.00
		Mean IOP ± SD (mmHg)	11.25 ± 1.16	7.37 ± 1.19	8.50 ± 0.93	9.87 ± 1.25	9.37 ± 1.85	9.50 ± 0.93	9.50 ± 1.85	9.87 ± 2.17
Group A	Left eye	Range	7.00–11.00	5.00–11.00	7.00–10.00	6.00–9.00	5.00–12.00	5.00–9.00	5.00–9.00	5.00–9.00
		Median	8.50	7.50	8.50	7.50	9.00	7.50	7.00	7.00
		Mean IOP ± SD (mmHg)	8.75 ± 1.28	7.87 ± 2.23	8.62 ± 1.06	7.37 ± 1.06	9.00 ± 2.45	7.12 ± 1.64	7.00 ± 1.19	7.00 ± 1.31
	Right eye	Range	7.00–12.00	4.00–9.00	5.00–10.00	7.00–10.00	6.00–12.00	7.00–10.00	7.00–10.00	8.00–10.00
		Median	9.00	7.50	9.00	8.50	10.00	9.00	9.00	9.00
		Mean IOP ± SD (mmHg)	9.00 ± 1.41	7.25 ± 1.83	8.37 ± 1.60	8.62 ± 1.06	9.87 ± 2.17	8.87 ± 0.99	8.50 ± 1.07	8.87 ± 0.64
Time			5:30 AM (baseline)	6:00 AM	7:00 AM	8:00 AM	9:00 AM	12:00 PM	15:00 PM	18:00 PM

IOP reduced significantly in the right eye post‐instillation of latanoprost in Group A at 6:00 (−1.75 ± 1.49 mmHg, *p*‐value = 0.013), whereas there was no significant IOP reduction after 6:00 (*p*‐value >0.05). In Group B, IOP reduced non‐significantly at 6:00 in the right and left eyes by −3.87 ± 0.99 (*p*‐value = 0.084) and −1.12 ± 1.36 (*p*‐value = 0.051) mmHg, respectively. However, the IOP reduction was significant in the right eye in Group B at 7:00 (−2.75 ± 1.49 mmHg, *p*‐value = 0.011) and 8:00 (−1.37 ± 0.92 mmHg, *p*‐value = 0.047) and remained low at 12:00 and 15:00 (−1.75 ± 1.03 mmHg, *p*‐value = 0.017 and −1.75 ± 1.16 mmHg, *p*‐value = 0.023, respectively).

There was a significant reduction of IOP in the left eye due to the systemic absorption of latanoprost in Group A at 8:00, 12:00, 15:00 and 18:00 by −1.37 ± 1.41, −1.62 ± 1.06, −1.75 ± 0.71 and −1.75 ± 1.83 mmHg (*p*‐value <0.05); and at 7:00 in Group B (−1.37 ± 0.92 mmHg, *p*‐value = 0.016). The reduction of IOP values from the baseline and the *p*‐values associated with them in Groups A and B are demonstrated by negative values in Table 2.

**TABLE 2:** The changes associated with intraocular pressure (IOP) (mmHg) (mean values ± standard deviation [SD]) of the guinea pigs’ right and left eye from the baseline after the administration of 0.005% latanoprost in Groups A and B during the 12 + 0.5 h of the experiment.

Time	Group A				Group B			
	Right eye IOP (mean IOP ± SD)–baseline (mmHg)	*p*‐value	Left eye IOP (mean IOP ± SD)–baseline (mmHg)	*p*‐value	Right eye IOP (mean IOP ± SD)–baseline (mmHg)	*p*‐value	Left eye IOP (mean IOP ± SD)–baseline (mmHg)	*p*‐value
6:00	−1.75 ± 1.49	**0.013**	−0.87 ± 2.29	0.317	−3.87 ± 0.99	0.084	−1.12 ± 1.36	**0.051**
7:00	−0.62 ± 2.13	0.399	−0.12 ± 1.46	1.000	−2.75 ± 1.49	**0.011**	−1.37 ± 0.92	**0.016**
8:00	−0.37 ± 1.92	0.598	−1.37 ± 1.41	**0.028**	−1.37 ± 0.92	**0.047**	−0.25 ± 1.39	0.629
9:00	0.87 ± 2.10	0.277	0.25 ± 3.06	0.824	−1.87 ± 1.36	0.063	−0.62 ± 2.06	0.420
12:00	−0.12 ± 1.55	0.914	−1.62 ± 1.06	**0.017**	−1.75 ± 1.03	**0.017**	−0.75 ± 1.39	0.194
15:00	−0.50 ± 1.77	0.414	−1.75 ± 0.71	**<0.001**	−1.75 ± 1.16	**0.023**	−0.75 ± 1.49	0.197
18:00	−0.12 ± 1.46	0.854	−1.75 ± 1.83	**0.031**	−1.37 ± 1.99	0.088	−0.87 ± 1.46	0.133

*Note*: Significant *p*‐values have been indicated in bold font.

**FIGURE 1:**
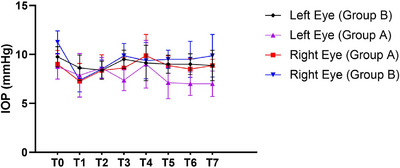
The effect of 0.005% latanoprost administration on intraocular pressure (IOP) (mmHg) in guinea pigs’ right and left eyes from Group A (exposed to 12 h of light; purple and red symbols) and Group B (kept in darkness for 12 h; black and blue symbols) at eight measurement points (T0–T7) over a 12 + 0.5 h experimental period. Data are presented as mean ± standard deviation (SD). Error bars indicate SD for each time point. IOP fluctuations were assessed to determine the impact of light exposure on latanoprost efficacy.

The graph in Figure 1 illustrates the pattern of right and left eye IOP fluctuations in Groups A and B (GraphPad Prism 8 software, version 8.2.1.441). The intragroup comparison (Mann‐Whitney *U* test) between the right and left eyes in Groups A and B demonstrated a significant difference between the IOP value at 5:30 prior to drop instillation and at 8:00 in both eyes, as well as at 12:00, 15:00 and 18:00 in the left eyes (Table 3). Moreover, the IOP values differed significantly between the right and left eyes (intergroup comparison by Wilcoxon Signed Rank test) at 6:00, 9:00, 12:00 and 18:00 in Group A and at 5:30, 6:00, 12:00 and 18:00 in Group B (Table 3).

**TABLE 3:** The *p*‐values associated with the comparison of the right and left eyes’ intraocular pressure (IOP) after the administration of 0.005% latanoprost between the Group A and B (intragroup comparison using Mann‐Whitney *U* test) and between each group (intergroup comparison using Wilcoxon signed rank test) at 8 measurement points (T0–T7) during the 12 + 0.5 h of the experiment in 16 healthy male guinea pigs.

Measurement points	Time	Intragroup comparison (Mann–Whitney *U* test)		Intergroup comparison (Wilcoxon signed rank test)	
		*p*‐value (R)	*p*‐value (L)	*p*‐value (Group A)	*p*‐value (Group B)
T0	5:30	**0.004**	0.108	0.094	**0.026**
T1	6:00	0.874	0.425	**0.023**	**0.011**
T2	7:00	0.721	0.721	0.726	0.705
T3	8:00	**0.049**	**0.001**	0.081	0.285
T4	9:00	0.627	0.909	**<0.001**	0.649
T5	12:00	0.279	**0.015**	**0.017**	**0.046**
T6	15:00	0.328	**0.003**	0.075	0.317
T7	18:00	0.328	**0.021**	**0.011**	**0.034**

*Note*: Significant *p*‐values have been indicated in bold font.

Abbreviation: SD, standard deviation.

## 5 Discussion

Latanoprost, first introduced into the market in 1996, is an ester PGF2‐alpha prostaglandin analogue for the treatment of glaucoma, which reduces IOP by its effect on the collagens within the uveoscleral pathway (Russo et al. 2008; Stolz and Alm 2014). This drug has been previously shown to be effective in IOP reduction in humans (Larsson 2001; Orzalesi et al. 2003, 2000; Quaranta et al. 2006), and significant IOP reduction by 22%–33% was achieved in glaucoma patients (IOP > 21 mmHg) over long‐term treatment (Perry et al. 2003). The effect of latanoprost on IOP has been investigated on several other animal species as well, such as monkeys (Serle et al. 1998; Wang et al. 2007), dogs (Gelatt and MacKay 2001; Sarchahi et al. 2012), cats (McDonald et al. 2016; Studer et al. 2000) and rodents (Aihara et al. 2002; Husain et al. 2006; Ota et al. 2005).

Moreover, an animal's exposure to light affects the 24‐h pattern of IOP variations and the control of circadian rhythm (Buijs and Kalsbeek 2001; Cahill and Menaker 1989; R. Y. Moore and Silver 1998). IOP fluctuations have been previously described in several nocturnal species, such as guinea pig, where IOP values under topical anaesthesia were lower during the light phase, measured by rebound and applanation tonometry (3.68 and 13.37 mmHg, respectively), compared to the dark phase (8.12 and 20.62 mmHg, respectively) (Ansari‐Mood et al. 2016).

Moreover, IOP was higher during the dark phase (19.3 ± 1.9 mmHg) compared to the light phase (31.3 ± 1.3 mmHg) in mice under a 12 light/12 dark regime by Tonopen XL tonometry (C. G. Moore et al. 1996). The work of Rowland et al. (1981) demonstrated the pattern of diurnal IOP fluctuations in rabbits, in which IOP was higher by 10 mmHg in the dark phase, which was supported by other studies (Gregory et al. 1985). Due to the effect of light on IOP in normal conditions, the efficacy of ocular agents might be altered by the photic inputs. The effect of prostaglandin hypotensive agents on IOP has been investigated before in guinea pigs, in which IOP reduction was greater in darkness (Armin et al. 2023).

In our experiment, we investigated the effect of latanoprost on IOP in different light and dark conditions. The IOP reduction in the treated eyes was greater in the guinea pigs in the dark after latanoprost instillation at 7:00 and 8:00 (−2.75 ± 1.49 and −1.37 ± 0.92 mmHg, respectively, *p*‐value <0.05) compared to those animals in the light (−0.62 ± 2.13 and −0.37 ± 1.92 mmHg, *p*‐value >0.05). However, the IOP reduction was not significant in the treated eyes of the animals in the light after 7:00 until the end of the experiment (Table 2).

The systemic absorption of latanoprost, a prostaglandin F2α analogue, occurs primarily through the conjunctival and nasolacrimal pathways, allowing the drug to enter systemic circulation after topical application. Pharmacokinetic studies of prostaglandin analogues indicate that latanoprost is rapidly hydrolysed to its active form, latanoprost acid, which can reach the bloodstream and exert effects on both treated and untreated eyes (Sjöquist and Stjernschantz 2002). This phenomenon, known as a contralateral effect, has been observed in various species where IOP reduction occurs in the untreated eye due to systemic distribution (Alvan et al. 1980).

Differences between the light and dark groups in IOP response may be attributed to circadian variations in aqueous humour dynamics, as studies suggest that prostaglandin analogues exhibit enhanced efficacy during the nocturnal phase when aqueous humour production is reduced (Doshi et al. 2009). In mammals, light exposure influences melatonin secretion and autonomic regulation of IOP, potentially modifying the pharmacokinetics and pharmacodynamics of latanoprost (Zhang et al. 2024). In the dark group, altered ocular physiology, including changes in uveoscleral outflow and receptor sensitivity, may result in a differential response to latanoprost compared to the light‐exposed group. These findings align with previous studies on prostaglandin analogues, emphasizing the importance of systemic absorption and circadian influences in the interpretation of IOP‐lowering effects.

Although significant IOP reduction was observed in the untreated eyes of the guinea pigs in both light and darkness conditions due to systemic absorption, this effect was more prominent in the light group, in which IOP decreased significantly at 8:00, 12:00, 15:00 and 18:00 (*p*‐value <0.05) (Table 2). It remains unclear which physiological mechanisms are involved to explain this difference. Moreover, data from IOP measurements taken at baseline revealed that in both groups, the right eye's IOP values were marginally higher than the left eye's due to the effect of measurement order, which has been found to affect IOP readings because of ocular squeezing (Pekmezci et al. 2011).

In guinea pigs, the more pronounced reduction of IOP observed during periods of darkness can be due to the increased activity of rod photoreceptors, which are more active in low‐light conditions. This heightened activity can influence retinal signalling pathways that modulate IOP, as observed earlier in rats (Nicou and Passaglia 2024).

The circadian clock, governed by the suprachiasmatic nucleus, orchestrates daily rhythms in various physiological processes, including IOP regulation. In nocturnal animals like guinea pigs, the circadian system may adjust IOP‐lowering mechanisms to align with their active phases during darkness. Additionally, the autonomic nervous system, comprising sympathetic and parasympathetic branches, plays a pivotal role in ocular physiology. During darkness, alterations in autonomic tone, such as reduced sympathetic activity, can lead to decreased aqueous humour production or increased outflow, culminating in lower IOP (Hoyng 1981).

As latanoprost does not have a significant effect on the permeability of the blood‐aqueous barrier, IOP returns to normal by a few weeks after the stop of therapy; thus, latanoprost is considered safe for glaucoma patients with chronic ocular hypertension (Lindén et al. 1997). However, if the patient already has ocular hypertension, this may be taken into consideration during therapy. Additionally, combining medications or shortening the time between doses may help lower IOP.

Systemic absorption of latanoprost following topical ocular administration is minimal due to its rapid metabolism in the plasma. This characteristic suggests a low potential for systemic side effects, allowing for its safe use in clinical settings. However, to further minimize systemic absorption, it is recommended to apply nasolacrimal occlusion or gently close the eyelid after administration (https://Periop‐Handbook.Ukclinicalpharmacy.Org/Drug/Latanoprost/).

In guinea pigs, studies have demonstrated that topical application of latanoprost effectively reduces IOP without significant systemic absorption, indicating that standard dosing regimens are appropriate for achieving desired ocular effects while minimizing systemic exposure (Dhungel et al. 2024).

One limitation of this study was the small sample size (*n* = 8 per group), which may reduce statistical power and limit the generalizability of the findings. A larger sample size would provide more robust data and minimize individual variability. Additionally, the study exclusively used male guinea pigs, which prevents the assessment of potential sex‐based differences in IOP regulation and latanoprost efficacy. Female guinea pigs may exhibit different hormonal influences on IOP, particularly under varying light conditions, as oestrogen and other sex hormones are known to affect aqueous humour dynamics. Additionally, standardized and validated restraint techniques, along with stress assessment, would improve data reliability. Addressing these limitations in future studies would enhance the accuracy and applicability of the findings.

## 6 Conclusions

The variations in the IOP fluctuations post‐instillation of 0.005% latanoprost were observed in both groups; however, the reduction in IOP was more pronounced in guinea pigs kept in darkness compared to those exposed to light. Therefore, the circadian pattern of IOP fluctuations should be considered as an influential factor in guinea pig glaucoma management.

## Author Contributions

**Arghavan Armin**: data gathering, animal handling, statistical analysis, manuscript writing. **Farnoosh Arfaee**: experimental design, conceptualization, manuscript review, data interpretation and statistical analysis.

## Ethics Statement

The ethics committee of the Science and Research Branch of the Islamic Azad University (IR.IAU.SRB.REC.1399.078) and the ARVO Statement for the Use of Animals in Ophthalmic and Vision Research approved the current study.

## Conflicts of Interest

The authors declare no conflicts of interest.

## Peer Review

The peer review history for this article is available at https://www.webofscience.com/api/gateway/wos/peer‐review/10.1002/vms3.70560.

## Data Availability

The data that support the findings of this study are available on request from the corresponding author. The data are not publicly available due to privacy or ethical restrictions.

## References

[vms370560-bib-0002] Aihara, M. , J. D. Lindsey , and R. N. Weinreb . 2002. “Reduction of Intraocular Pressure in Mouse Eyes Treated With Latanoprost.” Investigative Ophthalmology & Visual Science 43, no. 1: 146–150. 11773025

[vms370560-bib-0003] Aihara, M. , J. D. Lindsey , and R. N. Weinreb . 2003. “Twenty‐Four‐Hour Pattern of Mouse Intraocular Pressure.” Experimental Eye Research 77, no. 6: 681–686. 14609556 10.1016/j.exer.2003.08.011

[vms370560-bib-0004] Alvan, G. , B. Calissendorff , P. Seideman , K. Widmark , and G. Widmark . 1980. “Absorption of Ocular Timolol1.” Clinical Pharmacokinetics 5, no. 1: 95–100. 10.2165/00003088-198005010-00004. 7363532

[vms370560-bib-0005] Ansari‐Mood, M. , S. Mehdi‐Rajaei , R. Sadjadi , M. Selk‐Ghaffari , and D. L. Williams . 2016. “Twenty‐Four–Hour Measurement of Intraocular Pressure in Guinea Pigs (*Cavia porcellus*).” Journal of the American Association for Laboratory Animal Science 55, no. 1: 95–97. 26817986 PMC4747017

[vms370560-bib-0006] Armin, A. , F. Arfaee , S. Ozmaie , and A. Asghari . 2023. “The Evaluation of the Effect of Tafluprost on the Intraocular Pressure of Healthy Male Guinea Pigs Under Different Light‐and‐Darkness Regimes.” Veterinary Medicine and Science 9, no. 3: 1172–1178. 10.1002/vms3.1082. 36757117 PMC10188098

[vms370560-bib-0007] Bertolucci, C. , E. Giudice , F. Fazio , and G. Piccione . 2009. “Circadian Intraocular Pressure Rhythms in Athletic Horses Under Different Lighting Regime.” Chronobiology International 26, no. 2: 348–358. 10.1080/07420520902751035. 19212846

[vms370560-bib-0008] Blumberg, D. , N. Congdon , H. Jampel , et al. 2007. “The Effects of Sevoflurane and Ketamine on Intraocular Pressure in Children During Examination Under Anesthesia.” American Journal of Ophthalmology 143, no. 3: 494–499. 17317393 10.1016/j.ajo.2006.11.061

[vms370560-bib-0009] Buijs, R. M. , and A. Kalsbeek . 2001. “Hypothalamic Integration of central and Peripheral Clocks.” Nature Reviews Neuroscience 2, no. 7: 521–526. 11433377 10.1038/35081582

[vms370560-bib-0010] Cahill, G. M. , and M. Menaker . 1989. “Effects of Excitatory Amino Acid Receptor Antagonists and Agonists on Suprachiasmatic Nucleus Responses to Retinohypothalamic Tract Volleys.” Brain Research 479, no. 1: 76–82. 2538206 10.1016/0006-8993(89)91337-1

[vms370560-bib-0011] Devlin, P. F. , and S. A. Kay . 2001. “Circadian Photoperception.” Annual Review of Physiology 63, no. 1: 677–694. 10.1146/annurev.physiol.63.1.677. 11181972

[vms370560-bib-0012] Dhungel, P. , S. A. Vyas , and C. F. Wildsoet . 2024. “The Comparative Effectiveness of Latanoprostene and Latanoprost in Lowering IOP in Young Guinea Pigs, a Common Myopia Model.” Investigative Ophthalmology & Visual Science 65, no. 7: 1174–1174.

[vms370560-bib-0013] Di, Y. , X.‐M. Luo , T. Qiao , and N. Lu . 2017. “Intraocular Pressure With Rebound Tonometry and Effects of Topical Intraocular Pressure Reducing Medications in Guinea Pigs.” International Journal of Ophthalmology 10, no. 2: 186–190. 28251075 10.18240/ijo.2017.02.02 PMC5313539

[vms370560-bib-0014] Doshi, A. B. , J. H. K. Liu , and R. N. Weinreb . 2009. “Circadian Changes in Intraocular Pressure.” In Glaucoma, edited by F. Grehn and R. Stamper , 23–28. Springer. 10.1007/978-3-540-69475-5_3.

[vms370560-bib-0015] Drance, S. M. 1963. “Diurnal Variation of Intraocular Pressure in Treated Glaucoma: Significance in Patients With Chronic Simple Glaucoma.” Archives of Ophthalmology 70, no. 3: 302–311. 14048787 10.1001/archopht.1963.00960050304004

[vms370560-bib-0016] Gelatt, K. N. , and E. O. MacKay . 2001. “Effect of Different Dose Schedules of Latanoprost on Intraocular Pressure and Pupil Size in the Glaucomatous Beagle.” Veterinary Ophthalmology 4, no. 4: 283–288. 10.1046/j.1463-5216.2001.00201.x. 11906665

[vms370560-bib-0001] Google Search . n.d. Retrieved March 11, 2025. https://periop‐handbook.ukclinicalpharmacy.org/drug/latanoprost/.

[vms370560-bib-0017] Green, C. B. , J. S. Takahashi , and J. Bass . 2008. “The Meter of Metabolism.” Cell 134, no. 5: 728–742. 18775307 10.1016/j.cell.2008.08.022 PMC3760165

[vms370560-bib-0018] Gregory, D. S. , D. G. Aviado , and M. L. Sears . 1985. “Cervical Ganglionectomy Alters the Circadian Rhythm of Intraocular Pressure in New Zealand White Rabbits.” Current Eye Research 4, no. 12: 1273–1280. 10.3109/02713688509017687. 4085255

[vms370560-bib-0019] Hoyng, P. F. J. 1981. “The Autonomic Nervous System and the Intraocular Pressure.” In Pharmacological Denervation and Glaucoma, edited by P. F. J. Hoyng , 7–21. Springer. 10.1007/978-94-009-8674-9_2.

[vms370560-bib-0020] Husain, S. , N. A. Whitlock , D. S. Rice , and C. E. Crosson . 2006. “Effects of Latanoprost on Rodent Intraocular Pressure.” Experimental Eye Research 83, no. 6: 1453–1458. 17027754 10.1016/j.exer.2006.08.004

[vms370560-bib-0021] Komáromy, A. M. , D. Bras , D. W. Esson , et al. 2019. “The Future of Canine Glaucoma Therapy.” Veterinary Ophthalmology 22, no. 5: 726–740. 10.1111/vop.12678. 31106969 PMC6744300

[vms370560-bib-0022] Larsson, L.‐I. 2001. “Intraocular Pressure Over 24 Hours After Repeated Administration of Latanoprost 0.005% or Timolol Gel‐Forming Solution 0.5% in Patients With Ocular Hypertension.” Ophthalmology 108, no. 8: 1439–1444. 11470697 10.1016/s0161-6420(01)00605-4

[vms370560-bib-0023] Lindén, C. , E. Nuija , and A. Alm . 1997. “Effects on IOP Restoration and Blood‐Aqueous Barrier After Long Term Treatment With Latanoprost in Open Angle Glaucoma and Ocular Hypertension.” British Journal of Ophthalmology 81, no. 5: 370–372. 9227201 10.1136/bjo.81.5.370 PMC1722192

[vms370560-bib-0024] Liu, J. H. , D. F. Kripke , R. E. Hoffman , et al. 1999. “Elevation of Human Intraocular Pressure at Night Under Moderate Illumination.” Investigative Ophthalmology & Visual Science 40, no. 10: 2439–2442. 10476816

[vms370560-bib-0025] McDonald, J. E. , J. A. Kiland , P. L. Kaufman , E. Bentley , N. M. Ellinwood , and G. J. McLellan . 2016. “Effect of Topical Latanoprost 0.005% on Intraocular Pressure and Pupil Diameter in Normal and Glaucomatous Cats.” Veterinary Ophthalmology 19, no. S1: 13–23. 10.1111/vop.12292. 26183373 PMC4755930

[vms370560-bib-0026] Moore, C. G. , E. C. Johnson , and J. C. Morrison . 1996. “Circadian Rhythm of Intraocular Pressure in the Rat.” Current Eye Research 15, no. 2: 185–191. 10.3109/02713689608997412. 8670727

[vms370560-bib-0027] Moore, R. Y. , and R. Silver . 1998. “Suprachiasmatic Nucleus Organization.” Chronobiology International 15, no. 5: 475–487. 10.3109/07420529808998703. 9787937

[vms370560-bib-0028] Nicou, C. M. , and C. L. Passaglia . 2024. “Effect of Ambient Lighting on Intraocular Pressure Rhythms in Rats.” Investigative Ophthalmology & Visual Science 65, no. 10: 16–16. 10.1167/iovs.65.10.16 PMC11314632 39115866

[vms370560-bib-0029] Ofri, R. 2002. “Intraocular Pressure and Glaucoma.” Veterinary Clinics: Exotic Animal Practice 5, no. 2: 391–406. 12170640 10.1016/s1094-9194(01)00004-4

[vms370560-bib-0030] Okur, S. , L. Yanmaz , M. Senocak , et al. 2023. “Comparison of Intraocular Pressure in New Zealand White Rabbits Measured Using Rebound and Applanation Tonometers and Four Different Methods of Physical Restraint.” New Zealand Veterinary Journal 71, no. 5: 251–258. 10.1080/00480169.2023.2224277. 37306141

[vms370560-bib-0031] Orzalesi, N. , L. Rossetti , A. Bottoli , E. Fumagalli , and P. Fogagnolo . 2003. “The Effect of Latanoprost, Brimonidine, and a Fixed Combination of Timolol and Dorzolamide on Circadian Intraocular Pressure in Patients With Glaucoma or Ocular Hypertension.” Archives of Ophthalmology 121, no. 4: 453–457. 12695241 10.1001/archopht.121.4.453

[vms370560-bib-0032] Orzalesi, N. , L. Rossetti , T. Invernizzi , A. Bottoli , and A. Autelitano . 2000. “Effect of Timolol, Latanoprost, and Dorzolamide on Circadian IOP in Glaucoma or Ocular Hypertension.” Investigative Ophthalmology & Visual Science 41, no. 9: 2566–2573. 10937568

[vms370560-bib-0033] Ostrin, L. A. , and C. F. Wildsoet . 2016. “Optic Nerve Head and Intraocular Pressure in the guinea Pig Eye.” Experimental Eye Research 146: 7–16. 26698659 10.1016/j.exer.2015.12.007 PMC4893889

[vms370560-bib-0034] Ota, T. , H. Murata , E. Sugimoto , M. Aihara , and M. Araie . 2005. “Prostaglandin Analogues and Mouse Intraocular Pressure: Effects of Tafluprost, Latanoprost, Travoprost, and Unoprostone, Considering 24‐Hour Variation.” Investigative Ophthalmology & Visual Science 46, no. 6: 2006–2011. 15914616 10.1167/iovs.04-1527

[vms370560-bib-0035] Pekmezci, M. , S. T. Chang , B. S. Wilson , M. O. Gordon , and A. M. Bhorade . 2011. “Effect of Measurement Order Between Right and Left Eyes on Intraocular Pressure Measurement.” Archives of Ophthalmology 129, no. 3: 276–281. 21402981 10.1001/archophthalmol.2011.33

[vms370560-bib-0036] Perry, C. M. , J. K. McGavin , C. R. Culy , and T. Ibbotson . 2003. “Latanoprost: An Update of Its Use in Glaucoma and Ocular Hypertension.” Drugs & Aging 20, no. 8: 597–630. 10.2165/00002512-200320080-00005. 12795627

[vms370560-bib-0037] Quaranta, L. , F. Gandolfo , R. Turano , et al. 2006. “Effects of Topical Hypotensive Drugs on Circadian IOP, Blood Pressure, and Calculated Diastolic Ocular Perfusion Pressure in Patients With Glaucoma.” Investigative Ophthalmology & Visual Science 47, no. 7: 2917–2923. 16799034 10.1167/iovs.05-1253

[vms370560-bib-0038] Reppert, S. M. , and D. R. Weaver . 2002. “Coordination of Circadian Timing in Mammals.” Nature 418, no. 6901: 935–941. 12198538 10.1038/nature00965

[vms370560-bib-0039] Rowland, J. M. , D. E. Potter , and R. J. Reiter . 1981. “Circadian Rhythm in Intraocular Pressure: A Rabbit Model.” Current Eye Research 1, no. 3: 169–173. 10.3109/02713688109001822. 7297102

[vms370560-bib-0040] Ruangvaravate, N. , K. Choojun , B. Srikulsasitorn , J. Chokboonpiem , D. Asanatong , and S. Trakanwitthayarak . 2020. “Ocular Surface Changes After Switching From Other Prostaglandins to Tafluprost and Preservative‐Free Tafluprost in Glaucoma Patients.” Clinical Ophthalmology 14: 3109–3119. 10.2147/OPTH.S264984. 33116362 PMC7548342

[vms370560-bib-0041] Russo, A. , I. Riva , T. Pizzolante , F. Noto , and L. Quaranta . 2008. “Latanoprost Ophthalmic Solution in the Treatment of Open Angle Glaucoma or Raised Intraocular Pressure: A Review.” Clinical Ophthalmology 2, no. 4: 897–905. 19668444 PMC2699817

[vms370560-bib-0042] Sarchahi, A. A. , N. Abbasi , and M. A. Gholipour . 2012. “Effects of an Unfixed Combination of Latanoprost and Pilocarpine on the Intraocular Pressure and Pupil Size of Normal Dogs.” Veterinary Ophthalmology 15, no. S1: 64–70. 10.1111/j.1463-5224.2011.00958.x. 22050700

[vms370560-bib-0043] Serle, J. B. , S. M. Podos , Y. Kitazawa , and R.‐F. Wang . 1998. “A Comparative Study of Latanoprost (Xalatan) and Isopropyl Unoprostone (Rescula) in Normal and Glaucomatous Monkey Eyes.” Japanese Journal of Ophthalmology 42, no. 2: 95–100. 9587840 10.1016/s0021-5155(97)00128-7

[vms370560-bib-0044] Sjöquist, B. , and J. Stjernschantz . 2002. “Ocular and Systemic Pharmacokinetics of Latanoprost in Humans.” Survey of Ophthalmology 47: S6–S12. 12204697 10.1016/s0039-6257(02)00302-8

[vms370560-bib-0045] Stolz, J. , and A. Alm . 2014. “Latanoprost in the Treatment of Glaucoma.” Clinical Ophthalmology 8: 1967–1985. 10.2147/OPTH.S59162. 25328381 PMC4196887

[vms370560-bib-0046] Studer, M. E. , C. L. Martin , and J. Stiles . 2000. “Effects of 0.005% Latanoprost Solution on Intraocular Pressure in Healthy Dogs and Cats.” American Journal of Veterinary Research 61, no. 10: 1220–1224. 11039551 10.2460/ajvr.2000.61.1220

[vms370560-bib-0047] Teng, C. , R. Gurses‐Ozden , J. M. Liebmann , C. Tello , and R. Ritch . 2003. “Effect of a Tight Necktie on Intraocular Pressure.” British Journal of Ophthalmology 87, no. 8: 946–948. 12881330 10.1136/bjo.87.8.946 PMC1771792

[vms370560-bib-0048] Wang, R.‐F. , D. J. Gagliuso , T. W. Mittag , and S. M. Podos . 2007. “Effect of 15‐Keto Latanoprost on Intraocular Pressure and Aqueous Humor Dynamics in Monkey Eyes.” Investigative Ophthalmology & Visual Science 48, no. 9: 4143–4147. 17724199 10.1167/iovs.07-0035

[vms370560-bib-0049] Weinreb, R. N. , T. Aung , and F. A. Medeiros . 2014. “The Pathophysiology and Treatment of Glaucoma: A Review.” JAMA 311, no. 18: 1901–1911. 24825645 10.1001/jama.2014.3192 PMC4523637

[vms370560-bib-0050] Zhang, J. , H. Zhou , Y. Cai , S. Yoshida , Y. Li , and Y. Zhou . 2024. “Melatonin: Unveiling the Functions and Implications in Ocular Health.” Pharmacological Research 205, no. 7: 107253. 38862072 10.1016/j.phrs.2024.107253

